# Treatment utilization and effectiveness of neoadjuvant chemotherapy comparing men and women diagnosed with breast cancer: a Swedish retrospective cohort study

**DOI:** 10.1007/s10549-023-07129-1

**Published:** 2023-10-09

**Authors:** Aglaia Schiza, Irma Fredriksson, Malin Sund, Antonios Valachis

**Affiliations:** 1 Science for Life Laboratory, Department of Immunology, Genetics and Pathology, Uppsala University, Dag Hammarskjoldsvag 20, 751 85 Uppsala, Sweden; 2 Department of Oncology, Uppsala University Hospital, 751 85 Uppsala, Sweden; 3 Department of Molecular Medicine and Surgery, Karolinska Institutet, Stockholm, Sweden; 4 Department of Breast, Endocrine Tumours and Sarcoma, Karolinska Comprehensive Cancer Center, Karolinska University Hospital, Stockholm, Sweden; 5 Department of Surgical and Perioperative Sciences/Surgery, Umeå University, Umeå, Sweden; 6 Department of Surgery, University of Helsinki and Helsinki University Hospital, Helsinki, Finland; 7 Department of Oncology, Faculty of Medicine and Health, Orebro University, Orebro, Sweden

**Keywords:** Early breast cancer, Neoadjuvant chemotherapy, Utilization of neoadjuvant chemotherapy, Effectiveness of neoadjuvant chemotherapy, Men, Pathologic complete response

## Abstract

**Purpose:**

Evidence supporting the use of neoadjuvant chemotherapy (NAC) in early breast cancer is based on studies mainly including women, whereas the utilization and effectiveness of NAC in men is less studied. The present study aimed to investigate the utilization and effectiveness of NAC in men and women with early breast cancer.

**Methods:**

Eligible patients were identified through the Swedish National Breast Cancer Quality Register, that includes all newly diagnosed breast cancer cases in Sweden from 2008 and onwards. For the treatment utilization analysis, all patients with stage I–III between 2008 and 2020 were included (n = 82,888), whereas for the effectiveness analysis the cohort was restricted to patients receiving NAC (n = 6487). For both analyses, multivariate logistic regression models were applied to investigate potential sex disparities in NAC utilization and effectiveness, adjusted for patient- and tumor characteristics.

**Results:**

In the NAC utilization analysis, 487 men and 82,401 women with stage I–III were included. No statistically significant difference between sexes in terms of NAC utilization was observed (adjusted Odds Ratio (adjOR): 1.135; 95% Confidence Interval (CI) 0.606–2.128) with an overall utilization rate of 4.9% in men compared to 7.8% in women. Among the 24 men and 6463 women who received NAC, the pathologic complete response (pCR) rates were 16.7% and 21.2%, respectively (adjOR: 1.141; 95% CI 0.141–9.238).

**Conclusion:**

The present study did not find any sex disparities in NAC utilization or effectiveness in terms of pCR. This supports the current recommendations of treating men with breast cancer with the same indications for NAC as women.

## Introduction

Breast cancer in men (male breast cancer; MBC) is a rare entity accounting for 0.5–1% of all breast cancer diagnoses and an estimated lifetime risk of 1:1000 [1]. Due to its rarity and lack of prospective dedicated MBC trials, the treatment guidelines are mainly based on extrapolation from randomized evidence from trials including mainly women with breast cancer (female breast cancer; FBC) [2].

The utilization of neoadjuvant chemotherapy (NAC) has steadily increased over the years due to its potential to de-escalate breast and axillary surgery as well as to provide an opportunity for response-based tailored adjuvant therapy [3]. There is solid evidence supporting the use of NAC in FBC, while the utilization and effectiveness of NAC in MBC is less studied [3, 4]. In fact, two retrospective studies have shown conflicting results regarding potential sex disparities on the effectiveness of NAC [5, 6], whereas one of them also showed a lower utilization of NAC in MBC compared to FBC [5].

Considering the limited and conflicting evidence on the role of NAC in MBC, the aims of the present nationwide, register-based, retrospective cohort study were to investigate the utilization of NAC in men and women with early breast cancer, and to compare the effectiveness of NAC between men and women in terms of pathologic complete response (pCR).

## Patients and methods

### Study design, data sources, participants and data collection

For this nationwide, register-based retrospective *cohort study*, all patients with stage I–III invasive breast cancer diagnosed in Sweden between January 1, 2008 and December 31, 2019 were identified through the National Quality Registry for breast cancer (Nationellt Kvalitetsregister för bröstcancer; NKBC). NKBC has a high coverage (99.8%) and data completeness ensuring the validity of data and the generalizability of the study results [7]. Using the ten-digit personal identity number, data from NKBC was linked to other national databases of interest to build the research database BCBaSe 3.0 (https://cancercentrum.se/samverkan/regional-cancer-centres/research-and-innovation/register-based-research-databases/). The study was performed in line with the principles of the Declaration of Helsinki. Approval was granted by the Regional Ethics Committee, Stockholm (Approval number: 2019–02610).

**Fig. 1:**
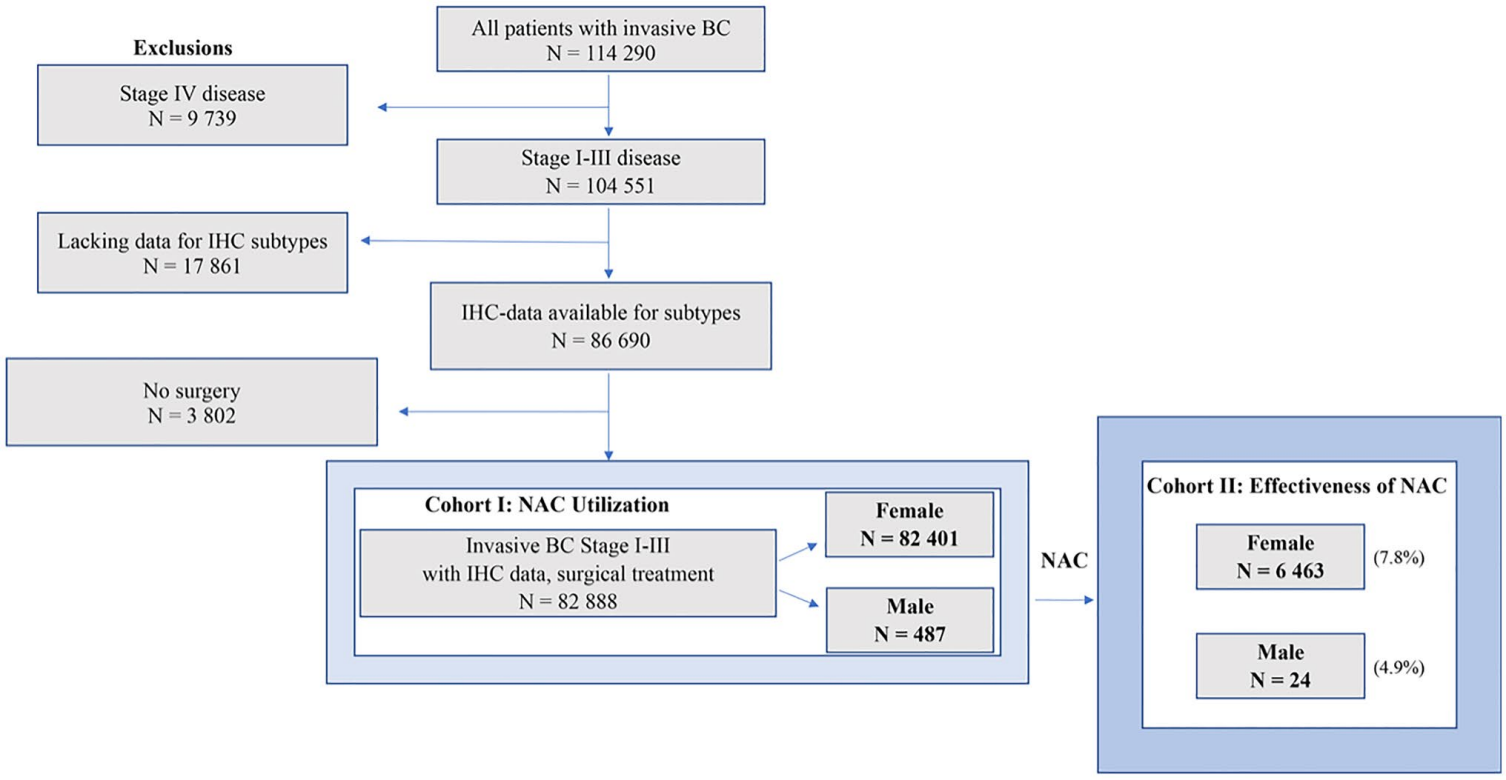
CONSORT diagram of study population. Cohort I: All patients registered in NKBC with stage I-III breast cancer who underwent surgery and had adequate information for IHC-subtyping. Cohort II: restricted only to patients who received neoadjuvant chemotherapy (NAC) (BcBASE 3.0, NKBC, 2008-2019).

All patients with stage I-III breast cancer in NKBC were included except those with lacking information on estrogen-receptor (ER) status, progesterone-receptor (PgR) status, HER2-status or information on preoperative or postoperative TNM stage. Patients who did not undergo surgery were also excluded. The number of patients treated with NAC but not underwent surgery were 91 females (1.4% among females with NAC) and 1 male (4.0% among males with NAC). The decision of no surgery has been considered unrelated to disease progression since all patients had a registered adjuvant therapy and none of the patients was registered with metastatic disease during the first three months from diagnosis (Fig. 1).

Patient demographics (age, educational level, household income, health care region at diagnosis), tumor characteristics (tumor size, histological grade, clinical stage, pathological stage, morphological type and surrogate molecular subtypes based on immunohistochemistry (IHC) status) and treatment characteristics (data on breast and axillary surgery, chemotherapy, radiotherapy and endocrine therapy) were collected.

### Outcomes and definitions

IHC-subtyping was used to classify tumors into three surrogate subtypes, namely luminal (ER or PgR-status ≥ 10%, HER2-negative), HER2-positive (any ER and PgR status, HER2-positive), and triple negative (ER and PgR status < 10% and HER2-negative) breast cancer.

For the research question on NAC utilization, patients were classified as treated with NAC if there was a treatment strategy including NAC, irrespective of chemotherapeutic agent used.

For the research question on pCR, we defined pCR as the absence of invasive breast cancer in the surgical specimens from breast and axillary lymph nodes.

### Statistical analysis

Data were summarized using descriptive statistics including numbers and percentages for categorical variables and median with range for continuous variables. Comparisons of patient characteristics between males and females were made by Pearson’s Chi-squared test, Fisher´s exact test, or Mann–Whitney test as appropriate. For the first research question on NAC utilization, a multivariable logistic regression analysis was performed to analyze the association between sex and the likelihood of receiving NAC while adjusting for prespecified patient- and tumor characteristics including age at diagnosis, educational level, household income, health care region at diagnosis, clinical T and N stage, morphological type, histological grade, and IHC-based subtype. For the second research question on pCR, a multivariable logistic regression analysis was conducted to evaluate the impact of sex on the odds of pCR, adjusted for prespecified parameters including age at diagnosis, clinical T and N stage, histological grade, and IHC-subtype. All *p* values reported were two-sided, and *p* values of < 0.05 were considered statistically significant. All statistical analyses were performed using the SPSS statistical package (IBM Corp. Released 2021. IBM SPSS Statistics for Windows, Version 28.0. Armonk, NY: IBM Corp).

## Results

### Characteristics of study cohort

In total, 114,290 patients with breast cancer were registered in NKBC between 2008 and 2020. Applying the inclusion and exclusion criteria, 82,888 patients (82,401 women and 428 men) with stage I-III breast cancer who underwent surgery and had adequate information for IHC-subtyping were identified. This cohort comprised the NAC utilization cohort. When the cohort was restricted only to patients who received NAC, 6487 breast cancer patients (6463 women and 24 men) were available for analyses related to the effectiveness of NAC. A flowchart diagram of patients’ selection process is shown in Fig. 1.

Table 1 summarizes patient, tumor and treatment characteristics in the NAC utilization cohort, by sex. Men with breast cancer were older, had a lower educational level, more advanced anatomical stage at diagnosis (both T and N stage), higher histological grade, fewer lobular carcinomas (1.5% vs. 13.8% in women) and a different IHC-subtype distribution (luminal 86.9% vs. 77.3%; HER2-positive 12.5% vs. 13.4%, triple negative 0.6% vs. 9.2%, respectively). Treatment patterns, including breast and axillary surgery as well as adjuvant therapeutic approaches followed the statistically significant differences in anatomical staging and IHC-subtype. Regarding NAC effectiveness cohort, 6463 women (7.8%) and 24 men (4.9%) received neoadjuvant chemotherapy, respectively. When comparing baseline characteristics of women and men treated with NAC, statistically significant differences regarding educational level, IHC-subtype, use of adjuvant endocrine therapy, as well as type of breast surgery were seen (Table 2). The utilization of NAC seems to be steadily increased over time for both males and females in Sweden as shown in Table 2.

**Table 1:** Summary of demographics, tumor-related variables, and treatment patterns for the utilization cohort; women and men with stage I-III breast cancer who underwent surgery and had adequate information for IHC-subtyping (BCBaSe 3.0/NKBC, 2008–2019)

Factors	Female (N = 82,401), n (%)	Male (N = 487), n (%)	p value
Age in yrs, median (range)	64 (19–99)	69 (29–94)	** < 0.001**
Calendar year at diagnosis			
2008–2011	22,001 (26.7)	108 (22.2)	**0.026**
2012–2015	26,780 (32.5)	154 (21.6)	
2016–2020	33,620 (40.8)	225 (46.2)	
Education Level			
Low ≦9 years	17,330 (21.3)	149 (30.8)	** < 0.001**
Intermediate 10–12 years	34,310 (42.1)	200 (41.3)	
High ≧13 years	29,790 (36.6)	135 (27.9)	
Household Income			
Q1	20,166 (24.6)	114 (23.5)	0.602
Q2	20,792 (25.4)	127 (26.2)	
Q3	20,545 (25.1)	132 (27.2)	
Q4	20,383 (24.9)	112 (23.1)	
Regions			
Northern	6822 (8.3)	47 (9.7)	0.519
Stockholm-Gotland	19,185 (23.4)	99 (20.4)	
Uppsala-Örebro	17,143 (20.9)	102 (21.0)	
South	12,965 (15.8)	79 (16.3)	
Southeast	8803 (10.7)	48 (9.9)	
Western (Halland)	17,142 (20.9)	11 (22.8)	
Clinical T stage			
T 0–1	54,155 (65.7)	263 (54.0)	** < 0.001**
T 2–4	27,893 (33.9)	221 (45.4)	
Clinical N stage			
cN +	10,246 (12.4)	108 (22.2)	** < 0.001**
cN-	71,881 (87.2)	378 (77.6)	
Histological grade			
Well differentiated (G1)	16,013 (19.4)	48 (9.9)	** < 0.001**
Moderately differentiated (G2)	40,137 (48.7)	246 (50.5)	
Poorly differentiated (G3)	21,346 (25.9)	171 (35.1)	
Subtype according to IHC			
Luminal	63,728 (77.3)	423 (86.9)	** < 0.001**
Her2 positive	11,059 (13.4)	61 (12.5)	
TNBC	7614 (9.2)	3 (0.6)	
Morphological subtype			
Ductal	61,414 (79.1)	429 (91.9)	** < 0.001**
Lobular	10,719 (13.8)	7 (1.5)	
Other	5503 (7.1)	31 (7.0)	
Breast surgery			
Breast conserving surgery	49,891 (60.5)	480 (98.6)	** < 0.001**
Mastectomy	32,510 (39.5)	7 (1.5)	
Axillary surgery			
Sentinel lymph node dissection (SLND)	55,566 (70.2)	265 (57.7)	** < 0.001**
Axillary lymph node dissection (ALND)	12,253 (15.5)	113 (24.6)	
SLND = > ALND	11,363 (14.4)	81 (17.6)	
Adjuvant chemotherapy			
Yes	23,029 (27.9)	163 (33.5)	**0.007**
No	59,372 (72.1)	324 (66.5)	
Adjuvant radiοtherapy			
Yes	50,513 (61.3)	156 (32.0)	** < 0.001**
No	31,888 (38.7)	331 (68.0)	
Adjuvant endocrine therapy			
Yes	53,368 (64.8)	383 (78.6)	** < 0.001**
No	29,033 (35.2)	104 (21.4)	
Neoadjuvant chemotherapy			
Yes	6463 (7.8)	24 (4.9)	**0.017**
No	75,938 (92.2)	463 (95.1)	

Bold text indicates a statistically significant difference with a p-value less than 0.05

**Table 2:** Summary of demographic and clinical variables as well as treatment modalities for female and male patients with breast cancer who received NAC (BCBaSe 3.0/NKBC, 2008–2019)

Factors	Female (N = 6463), n (%)	Male (N = 24), n (%)	p value
Age in yrs, median (range)	54 (21–83)	64.5 (41–84)	0.054
Calendar year at diagnosis			
2008–2011	866 (13.4)	3 (12.5)	0.462
2012–2015	1706 (26.4)	9 (37.5)	
2016–2020	3891 (60.2)	12 (50.0)	
Education Level	N = 6344	N = 23	**0.001**
Low ≦9 years	965 (15.2)	10 (43.5)	
Intermediate 10–12 years	2576 (40.6)	8 (34.8)	
High ≧13 years	2803 (44.2)	5 (21.7)	
Household Income	N = 6401	N = 24	0.405
Q4	2162 (33.8)	9 (37.5)	
Q3	1611 (25.2)	8 (33.3)	
Q2	1435 (22.4)	2 (8.3)	
Q1	1193 (18.6)	5 (20.8)	
Regions	N = 6420	N = 24	0.715
Northern	408 (6.4)	1 (4.2)	
Stockholm-Gotland	2476 (38.6)	10 (41.7)	
Uppsala-Örebro	794 (12.4)	1 (4.2)	
South	1224 (19.1)	7 (29.2)	
Southeast	615 (9.6)	2 (8.3)	
Western (Halland)	903 (14.1)	3 (12.5)	
Clinical T stage	N = 6463	N = 24	0.951
T 0–1	1066 (16.5)	4 (16.7)	
T 2–4	5370 (83.1)	20 (83.3)	
Clinical N stage	N = 6463	N = 24	0.407
cN +	3444 (53.3)	16 (66.7)	
cN-	2978 (46.1)	8 (33.3)	
Histological grade	N = 2394	N = 11	0.054
Well differentiated (G1)	266 (11.1)	1 (9.1)	
Moderately differentiated (G2)	1357 (56.7)	10 (90.9)	
Poorly differentiated (G3)	771 (32.2)	0 (0)	
Subtype according to IHC	N = 6463	N = 24	**0.001**
Luminal	2942 (45.4)	20 (83.3)	
Her2 positive	2147 (33.2)	4 (16.7)	
TNBC	1374 (21.3)	0 (0.0)	
Morphological	N = 2337	N = 11	0.349
Ductal	1961 (83.9)	11 (100)	
Lobular	262 (11.2)	0 (0.0)	
Other	114 (4.9)	0 (0.0)	
Breast surgery	N = 6463	N = 24	**0.001**
Breast conserving surgery	2132 (33)	0 (0.0)	
Mastectomy	4331 (67)	24 (100)	
Axillary surgery	N = 6271	N = 23	0.756
Sentinel lymph node dissection (SLND)	1575 (25.1)	5 (21.7)	
Axillary lymph node dissection (ALND)	3912 (62.4)	16 (69.6)	
SLND = > ALND	784 (12.5)	2 (8.7)	
Type of chemotherapy			
Anthracycline- and taxane-based	4858 (75.2)	18 (75.0)	0.817
Anthracycline-based	1004 (15.5)	3 (12.5)	
Taxane-based	601 (9.3)	3 (12.5)	
Anti-HER2 treatment			
Trastuzumab	1917 (29.7)	4 (16.7)	0.101
Adjuvant radiotherapy	N = 6463	N = 24	0.629
Yes	4854 (75.1)	17 (70.8)	
No	1609 (24.9)	7 (29.2)	
Adjuvant endocrine therapy	N = 6463	N = 24	** < 0.001**
Yes	3853 (55.4)	22 (91.7)	
No	2880 (44.6)	2 (8.3)	

Bold text indicates a statistically difference with a p-value less than 0.05

### Factors associated with NAC utilization patterns

Using multivariate logistic regression model, using the complete case analysis method, no statistically significant difference in NAC utilization between women and men was observed (Odds Ratio (OR): 1.135; 95% Confidence Interval (CI): 0.606–2.128). The total number of patients included in this model was 78,760. Factors associated with higher likelihood of NAC were: young age, high educational level, high household income, treatment in certain healthcare regions Stockholm/Gotland, South, or Southeast), high clinical T and N stage, high histological grade, HER2-positive and triple negative IHC-subtype and ductal histology (Table 3).

**Table 3:** In utilization cohort: multivariable logistic regression analyzing the association between receipt of NAC and clinical factors among patients with breast cancer (BCBaSe 3.0/NKBC, 2008–2019)

	OR	95% CI	95% CI	P-value
		Low	High	
Female versus male	1.135	0.606	2.128	0.692
Age	0.967	0.963	0.970	** < 0.001**
Educational Level				
High (≧13 years)	Ref			
Intermediate (10–12 years)	0.732	0.628	0.853	** < 0.001**
Low (≦9 years)	0.890	0.801	0.989	**0.030**
Household Income				
Q4 (High)	Ref			
Q3	0.708	0.624	0.803	** < 0.001**
Q2	0.676	0.591	0.773	** < 0.001**
Q1 (Low)	0.585	0.504	0.680	** < 0.001**
Healtcare regions				
Northern	Ref			
Stockholm Gotland	2.707	2.197	3.335	** < 0.001**
Uppsala-Örebro	0.826	0.657	1.038	0.101
South	2.119	1.698	2.645	** < 0.001**
Southeast	1.664	1.313	2.108	** < 0.001**
Western	0.813	0.647	1.023	0.077
Clinical T stage				
T1-2	Ref			
T3-4	4.151	1.782	9.667	** < 0.001**
Clinical N stage				
cN-	Ref			
cN +	1.888	1.039	3.432	**0.037**
Histological grade				
I	Ref			
II	4.305	3.587	5.167	** < 0.001**
III	4.049	3.575	4.586	** < 0.001**
Subtype according to IHC				
Luminal	Ref			
HER2-positive	3.077	2.714	3.489	** < 0.001**
Triple-negative	5.876	5.077	6.802	** < 0.001**
Morphological type				
Ductal	Ref			
Lobular	0.778	0.669	0.906	** < 0.001**
Other	0.667	0.530	0.838	** < 0.001**

The multivariate models were complete case analyses

Bold text indicates a statistically significant difference with a p-value less than 0.05

### Factors associated with pCR after NAC

No statistically significant difference in pCR rates were observed between women and men in the multivariate logistic regression analysis, using the complete case analysis method (OR: 1.141; 95% CI 0.141–9.238). The total number of patients included in this model was 6215. Factors associated with higher pCR rates were young age, high histologic grade, and HER2-positive IHC-subtype (Table 4).

**Table 4:** Multivariable logistic regression analyzing factors associated with complete pathologic response, among patients who received NAC for breast cancer (NKBC, 2008–2019)

	OR	95% CI	95% CI	P-value
		Low	High	
Female versus male	1.141	0.141	9.238	0.902
Age	0.989	0.981	0.997	**0.010**
Clinical T stage				
T1-2	Ref			
T3-4	1.227	0.142	10.612	0.853
Clinical N stage				
cN−	Ref			
cN +	0.693	0.180	2.675	0.595
Histological grade				
I	Ref			
II	1.989	1.191	3.320	**0.009**
III	4.623	2.743	7.793	** < 0.001**
Subtype according to IHC				
Luminal	Ref			
HER2-positive	2.774	2.134	3.607	** < 0.001**
Triple-negative	1.046	0.753	1.451	0.790

The multivariate models were complete case analyses

Bold text indicates a statistically significant difference with a p-value less than 0.05

## Discussion

Using nationwide, register-based data from Sweden, we found no evidence of sex disparities regarding utilization of NAC in breast cancer patients when analyses were adjusted for patient- and tumor characteristics. In addition, the effectiveness of NAC in terms of pCR seems to be similar between men and women with breast cancer, supporting the current recommendations on treating men with breast cancer with the same principles as women with regard to NAC indications.

Due to the rarity of MBC and subsequently the lack of prospective trials, potential differences in utilization and effectiveness of NAC between men and women have been investigated only through register-based studies.

Regarding utilization of NAC in men with breast cancer, Cao et al. analyzed data from the United States National Cancer Database (NCDB) between 2004 and 2016, and found that men with node positive (N +) disease were less likely to be treated with NAC when compared to women. Interestingly, Cao et al. found an underutilization of oncological treatment in men with breast cancer in general, a pattern not seen in the present study cohort. Breast cancer treatment practices may vary between countries, and may also have changed over time. Our study cohort included patients diagnosed during more recent years, when sex disparities in breast cancer treatment strategies have been acknowledged [8], thus leading to efforts to mitigate these disparities. Differences between study cohorts with regard to age (a higher proportion of older adults in our cohort) and stage (only patients with N + disease in NCDB cohort) could also explain the partly conflicting study results.

Also, with regard to NAC effectiveness in men compared to women with breast cancer, the current literature shows somewhat conflicting results. Cao et al. found similar pCR rate between men and women treated with neoadjuvant chemotherapy, as well as a comparable overall survival. Leone et al. found the odds for pCR in women compared to men to be nearly twice as high when studying patients diagnosed 2010 to 2016 from the same database as Cao. Interestingly, the difference in pCR rates observed in the latter study was mostly driven by differences in pCR within the luminal HER2-negative and luminal HER2-positive subgroups. Our results are in accordance with Cao et al., but differ from Leone et al. Although the number of included patients in certain subgroups in our study cohort was not large enough to enable subgroup analyses, we included this potential confounding factor into the multivariate model when the impact of sex on NAC effectiveness was analyzed. The lack of difference in pCR rates between men and women with breast cancer, in spite of the higher percentage of luminal breast cancer in men, could possibly be explained by different distribution of Luminal A/B tumors between men and women, i.e. a higher proportion of more high risk Luminal B tumors in men [9, 10]. Luminal B breast cancer is associated with higher pCR rates than Luminal A [11], possibly balancing the chance of pCR between the two cohorts. Another potential explanation of similar NAC effectiveness between men and women despite the dominance of luminal tumors in men could be a higher presence of an immunological-enriched tumor microenvironment in male luminal tumors [12], a condition that has been associated with improved pCR rates in all breast cancer subtypes [13].

The comparison of patient- and tumor-related characteristics between men and women with breast cancer confirmed some well-established differences between the sexes; older age at diagnosis, more advanced N-stage at diagnosis, the dominance of luminal subtype and the rarity of TNBC and lobular histology in men with breast cancer.

Considering NAC utilization in general, some study results deserve attention. Our study results confirm a tumor-driven approach regarding NAC utilization with a higher use in more advanced and biologically more aggressive disease, which is in line with the current evidence and clinical practice. On the other hand, our study results imply some socioeconomic and geographic inequalities with lower odds to receive NAC among patients with lower income, lower education and among those from specific regions. Although similar inequalities have been reported previously [14, 15], such disparities are not acceptable and a deeper understanding is necessary in an effort to eliminate healthcare-related inequalities.

An interesting finding in terms of factors associated with higher pCR rates was the association between young age and a higher pCR rate. This finding is in accordance with a pooled analysis from eight randomized trials, where younger patients had higher odds of pCR, thus supporting the notion that tumor biology in younger patients might be more aggressive also within subtypes and therefore more susceptible to chemotherapy [16].

The study has several limitations that need to be addressed. First, data on planned treatment was used rather than actual treatment given, as NKBC data on planned treatment have a higher validity than given treatment for the studied time period. As a result, however, a risk of misclassification between NAC and primary surgery for some patients does exist. Second, the duration of planned NAC is lacking. However, the Swedish guidelines have steadily and throughout the years, recommended the use of six cycles (q3w) of chemotherapy, similar to the recommendation for adjuvant chemotherapy. In our study cohort, we lacked information about dose-dense chemotherapeutic regimens. However, this strategy has been rather uncommon in Sweden during the study period and there is no reason to believe that there would be any sex disparity in using dose dense regimens that could impact the prognosis. The limited sample size for men with breast cancer in some specific subtypes is also a limitation as it precludes from relevant subgroup analysis. To mitigate this source of bias, we tried to adjust the multivariate analyses using parameters of potential interest as breast cancer subtype. One could argue that the exclusion of patients who did not undergo surgery can result in immortal-time bias if disease progression during NAC is the reason for no surgery. However, within the group of patients treated with NAC, the proportion of patients who did not undergo surgery was extremely low in both sexes and was considered unrelated to disease progression, thus eliminating the risk for immortal-time bias. Finally, the nature of collected data for the present study does not allow any information about the role of patient or clinician in treatment decision regarding the type and sequence of treatment strategy between sexes.

Acknowledging the relative limited number of men with breast cancer included in the analyses, the current study did not find any sex disparities either in the NAC utilization or effectiveness supporting the current recommendations on treating men with breast cancer similar to women with regard to indications for NAC. The observed socioeconomical and geographical disparities in NAC utilization deserves a deeper understanding before designing strategies to eliminate these inequalities towards an equitable access to breast cancer care.

## Data Availability

Data are available from register holders (Statistics Sweden, Swedish National Board of Health and Welfare, the Regional Cancer Center Stockholm Gotland) for researchers with relevant ethical approvals and who meet the criteria for access to confidential data. The data are not publicly available due to restrictions by Swedish and European law, in order to protect patient privacy.
